# Biomimetic Nanotherapies: Red Blood Cell Based Core–Shell Structured Nanocomplexes for Atherosclerosis Management

**DOI:** 10.1002/advs.201900172

**Published:** 2019-04-24

**Authors:** Yi Wang, Kang Zhang, Xian Qin, Tianhan Li, Juhui Qiu, Tieying Yin, Junli Huang, Sean McGinty, Giuseppe Pontrelli, Jun Ren, Qiwei Wang, Wei Wu, Guixue Wang

**Affiliations:** ^1^ Key Laboratory for Biorheological Science and Technology of Ministry of Education State and Local Joint Engineering Laboratory for Vascular Implants Bioengineering College of Chongqing University Chongqing 400030 China; ^2^ Division of Biomedical Engineering University of Glasgow Glasgow G12 8QQ UK; ^3^ Istituto per le Applicazioni del Calcolo – CNR Via dei Taurini 19 00185 Roma Italy; ^4^ Department of Radiation Oncology Massachusetts General Hospital Harvard Medical School Boston MA 02114 USA; ^5^ Department of Cancer Biology Dana‐Farber Cancer Institute and Department of Biological Chemistry and Molecular Pharmacology Harvard Medical School Boston MA 02115 USA

**Keywords:** atherosclerosis, biomimetic, mathematical modeling, nanocomplexes, targeted delivery

## Abstract

Cardiovascular disease is the leading cause of mortality worldwide. Atherosclerosis, one of the most common forms of the disease, is characterized by a gradual formation of atherosclerotic plaque, hardening, and narrowing of the arteries. Nanomaterials can serve as powerful delivery platforms for atherosclerosis treatment. However, their therapeutic efficacy is substantially limited in vivo due to nonspecific clearance by the mononuclear phagocytic system. In order to address this limitation, rapamycin (RAP)‐loaded poly(lactic‐*co*‐glycolic acid) (PLGA) nanoparticles are cloaked with the cell membrane of red blood cells (RBCs), creating superior nanocomplexes with a highly complex functionalized bio‐interface. The resulting biomimetic nanocomplexes exhibit a well‐defined “core–shell” structure with favorable hydrodynamic size and negative surface charge. More importantly, the biomimetic nature of the RBC interface results in less macrophage‐mediated phagocytosis in the blood and enhanced accumulation of nanoparticles in the established atherosclerotic plaques, thereby achieving targeted drug release. The biomimetic nanocomplexes significantly attenuate the progression of atherosclerosis. Additionally, the biomimetic nanotherapy approach also displays favorable safety properties. Overall, this study demonstrates the therapeutic advantages of biomimetic nanotherapy for atherosclerosis treatment, which holds considerable promise as a new generation of drug delivery system for safe and efficient management of atherosclerosis.

## 1 Introduction

Atherosclerosis is a progressive inflammatory disease characterized by the accumulation of lipids, immune cells, and fibrous elements in the artery wall. The disease is highly prevalent worldwide and is the leading cause of morbidity and mortality in industrialized countries.^1^, ^2^ Currently, both medication and surgical intervention are employed to treat atherosclerosis.^3^, ^4^ Unfortunately, general oral medication is limited to the treatment of early stage atherosclerosis. Due to its nonspecific distribution throughout the body, oral medication usually causes adverse effects, especially after long‐term treatment.^5^, ^6^ Although surgical interventions (e.g., stenting) are effective for the treatment of advanced atherosclerosis, such procedures are associated with side effects such as restenosis and late stent thrombosis, which hampers the long‐term success of surgical intervention.^7^

Nanotechnology promotes the specific delivery of therapeutic compounds and offers significant advantages over more traditional therapies, both in terms of efficacy and safety.^8^, ^9^, ^10^, ^11^ However, one shortcoming of conventional drug delivery nanosystems is the lack of site targeting. One of the important mechanisms responsible for limited targeting is that the phagocytic system of the human body recognizes nanoparticles as foreign substances and this leads to fast clearance.^12^, ^13^ Therefore, it is critical, yet difficult, to engineer nanoparticles with a biomimetic natural interface that evade the phagocyte system but still achieve specific targeting.^14^ The gold standard is to utilize synthetic hydrophilic and flexible polymers such as poly(ethylene glycol) (PEG) to modify the surface of the nanoparticles. Due to PEG's highly flexible and hydrophilic properties, a PEG coating creates a hydration layer to effectively reduce undesirable protein adsorption onto the surface, evade immune recognition and clearance, and then prolong the blood circulation time to enhance targeted drug delivery by EPR (enhanced permeability and retention) effects.^15^, ^16^ Although several PEG‐modified nanodelivery systems have achieved some success in the clinic,^17^, ^18^ it is increasingly being reported that the response of the immune system against the synthetic polymer, and the production of antibodies against PEG, potentially impair their performance in long‐term treatment.^19^, ^20^ More recently, cell membrane‐coated nanoparticles with highly complex functionalities for effective bio‐interfacing have been developed.^21^, ^22^, ^23^, ^24^ These membrane‐coated nanoparticles possess high biocompatibility and prolonged half‐life in the circulation, as well as exhibiting disease‐specific targeting. Therefore, such membrane‐coated nanomedicines have been employed in various research areas, including detoxification, vaccination, cardiovascular disease, and cancer.^14^, ^21^, ^25^, ^26^, ^27^ Various different types of membrane have been used to fabricate biomimetic nanoparticles. Among them, the red blood cell (RBC) membrane is an attractive choice to cloak nanoparticles because of its excellent biocompatibility, long half‐life in the circulation (≈120 days), and outstanding accessibility (the most abundant cell in blood).^28^, ^29^, ^30^ Indeed, studies have shown extended half‐life in blood, enhanced penetration and retention in tumor tissue when nanoparticles are cloaked with the RBC membrane.^26^, ^28^, ^29^, ^30^, ^31^ The EPR effect also exists in atherosclerotic lesions based on the leaky endothelium in inflammation and leaky microvessels in atherosclerotic plaque, allowing nanoparticles to permeate the vascular wall and accumulate within the pathological lesion.^32^, ^33^

**Figure 1:**
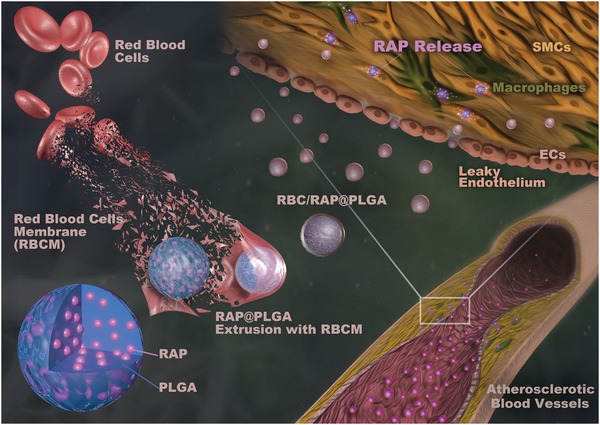
Illustrations displaying the preparation of RBC/RAP@PLGA for the treatment of atherosclerosis.

We hypothesized that coating nanoparticles with RBC membranes could enable them to evade the phagocytic system by incorporating the “natural” properties of the RBC membrane, thereby achieving a long half‐life in the circulation. We further hypothesized that loading these RBC membrane‐coated nanoparticles with an antiatherosclerotic compound would treat atherosclerosis more effectively than uncoated nanoparticles. In addition, poly(lactic‐*co*‐glycolic) acid (PLGA), a material approved by US food and drug administration (FDA), was selected as an excellent biocompatible and biodegradable material for efficient drug loading. Rapamycin (RAP), an inhibitor of the mammalian target of the rapamycin (mTOR) pathway, has been considered to be an effective antiatherosclerotic agent, in view of its multiple pharmacological activities including anti‐inflammation, antimigration, antiproliferation, and autophagy activation.^34^, ^35^ Therefore, the PLGA nanoparticles were loaded with RAP as a “core” structure (RAP@PLGA). We then coated the RAP@PLGA nanoparticles with RBC vesicles (RV), extracted from RBCs, to fabricate the RBC‐based “core–shell” structured nanocomplexes (**Figure** **1**). In this study, we have shown that this strategy enhances the half‐life of RAP@PLGA nanoparticles in the circulation. More importantly, we have shown that these biomimetic nanoparticles can accumulate within atherosclerotic plaques and efficiently inhibit the progression of atherosclerosis.

## 2 Results and Discussion

### 2.1 Fabrication and Characterization of RBC/RAP@PLGA

Efficient solubilization is critical for hydrophobic RAP to reach a sufficient dose in the atherosclerotic lesion. We, therefore, generated RAP@PLGA by encapsulating the hydrophobic RAP into the hydrophobic “core” of PLGA using a nanoprecipitation method.^36^ In contrast to the saturation solubility of RAP in water (only ≈2.6 µg mL^−1^),^37^ we were able to load 84.5 µg of RAP into 1 mL of PLGA aqueous solution (1 mg mL^−1^), thereby dramatically increasing its solubilization (more than 30 times greater than in water). As shown in Table S1 in the Supporting Information, drug loading efficiency (LE) and drug encapsulation efficiency (EE) of RAP@PLGA were 7.79% and 84.5%, respectively.

**Figure 2:**
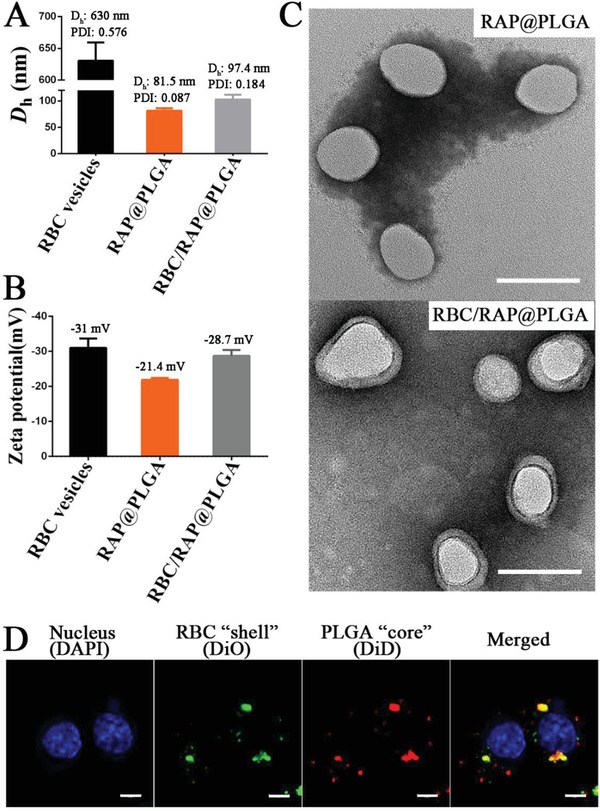
A) The *D*_h_ and B) zeta potential of RBC vesicles, RAP@PLGA and RBC/RAP@PLGA (*n* = 3, mean ± SD). C) TEM images of RAP@PLGA and RBC/RAP@PLGA (scale bar = 100 nm). D) CLSM images of RBC/DiD@PLGA internalization by cells, the nucleus (blue), RBC “shell” (green) and PLGA “core” (red) (scale bar = 5 µm).

Next, we sought to produce RBC/RAP@PLGA. RAP@PLGA was wrapped in the extracted RBC vesicles using a co‐extrusion method.^30^, ^38^ We first examined the hydrodynamic diameters (*D*_h_) of RBC vesicles, RAP@PLGA and RBC/RAP@PLGA by dynamic light scattering (DLS). As shown in **Figure** **2**A, coating with RBC vesicles increased the mean *D*_h_ from 81.5 ± 4.5 nm (RAP@PLGA, Polydispersity index (PDI): 0.087) to 97.4 ± 2.4 nm (RBC/RAP@PLGA, PDI: 0.184). This increase in *D*_h_ is consistent with the wrapping of the particles with RBC membranes, whose normal bilayer thickness is 7–8 nm.^26^, ^31^ Despite the fact that the *D*_h_ of RBC vesicles was 630 ± 28.6 nm (PDI: 0.576), the *D*_h_ of RBC/RAP@PLGA was close to that of RAP@PLGA, suggesting that the RBC membrane was tightly wrapped around the RAP@PLGA particles. Both RAP@PLGA and RBC/RAP@PLGA exhibited homogeneous dispersity (Figure S1, Supporting Information). Moreover, RBC/RAP@PLGA showed a relatively constant hydrodynamic diameter after long‐term storage at room temperature, indicating its favorable stability properties (Figure S2, Supporting Information). In addition, surface zeta potential analysis showed that RAP@PLGA has a zeta potential of −21.4 ± 0.6 mV, whereas RBC/RAP@PLGA (−28.7 ± 1.7 mV) and RBC vesicles (−31.0 ± 2.7 mV) have similar values (Figure 2B). We further analyzed the morphologies of RAP@PLGA and RBC/RAP@PLGA using transmission electron microscope (TEM). Both RAP@PLGA and RBC/RAP@PLGA showed a uniform sphere morphology. Moreover, RBC/RAP@PLGA displayed a clear core–shell nanostructure with a RAP@PLGA “core” and a RBC membrane “shell” (Figure 2C). These results confirm that the RBC membrane was successfully coated on the surface of RAP@PLGA, indicating successful fabrication of RBC/RAP@PLGA. To further verify the integrity of the core–shell nanoparticles, we loaded the PLGA core with hydrophobic DiD fluorophore and labeled the RBC membrane using DiO. We then incubated RAW264.7, a murine macrophage cell line, with these dual‐fluorophore‐labeled nanoparticles. The resulting fluorescent images showed that most of the DiD signal (red, representing the PLGA “core”) was co‐localized with the DiO signal (green, representing the RBC membrane “shell”), indicating an intact “core–shell” structure, even after cell internalization (Figure 2D). Moreover, because of the exclusive distribution of glycanprotein on the outside of cell membranes, the orientation of the RBC membrane on the surface of the nanoparticles could be evaluated by quantification of glycanprotein.^38^, ^39^ As shown in Figure S3 in the Supporting Information, the average content of glycoprotein on RBC/RAP@PLGA particles is around 93.26% of the equivalent amount in free RBC vesicles. This quantification suggests that glycoproteins are strongly retained on the outside surface of the RBC/RAP@PLGA particles, confirming the “right‐side‐out” orientation of the RBC membrane on the nanoparticles.

The release kinetics of RAP from RAP@PLGA nanoparticles and RBC/RAP@PLGA nanoparticles were investigated in PBS (pH 7.4) solution to simulate the physiological environment. After 72 h incubation in PBS, 35.96% of RAP was released from RBC/RAP@PLGA nanoparticles, while 38.52% of RAP was released from the RAP@PLGA nanoparticles. When compared with the RAP@PLGA nanoparticles, RBC/RAP@PLGA nanoparticles showed a slightly slower RAP release profile (Figure S4, Supporting Information), which may be ascribed to the additional cell membrane bilayer acting as a diffusion barrier. In conjunction with the experiments, we developed and numerically solved a dissolution–diffusion mathematical model of the drug release process (see Section 4 for full details of the model equations and parameters). We first simulated drug release from RAP@PLGA nanoparticles and found an excellent agreement between the model and the experimental data. Using the least squares method, the model was found to best‐fit the data with a Damköhler number of *Da* = 1396, corresponding to a diffusion coefficient of order 10^−18^ m^2^ s^−1^, and a normalized solubility of $\bar{S}=\;1.66\;\times 10^{-5}$ (Figure S5, Supporting Information). Using the same values of *Da* and $\bar{S},$ we then simulated drug release from RBC/RAP@PLGA nanoparticles (Figure S6, Supporting Information). We were able to capture the experimental data very well when the RBC were described as a thin membrane acting as a diffusion barrier, providing additional resistance to drug release. The best‐fit was found for a normalized membrane resistance of Γ = 173. These simulations confirm that drug release from RAP@PLGA and RBC/RAP@PLGA nanoparticles is well described by a dissolution–diffusion mechanism. In general, the RAP release profile from RBC/RAP@PLGA nanoparticles suggested their good potential to be used for sustained drug release.

### 2.2 RBC/RAP@PLGA Nanoparticles Display Immune‐Evasive Properties In Vitro and In Vivo

**Figure 3:**
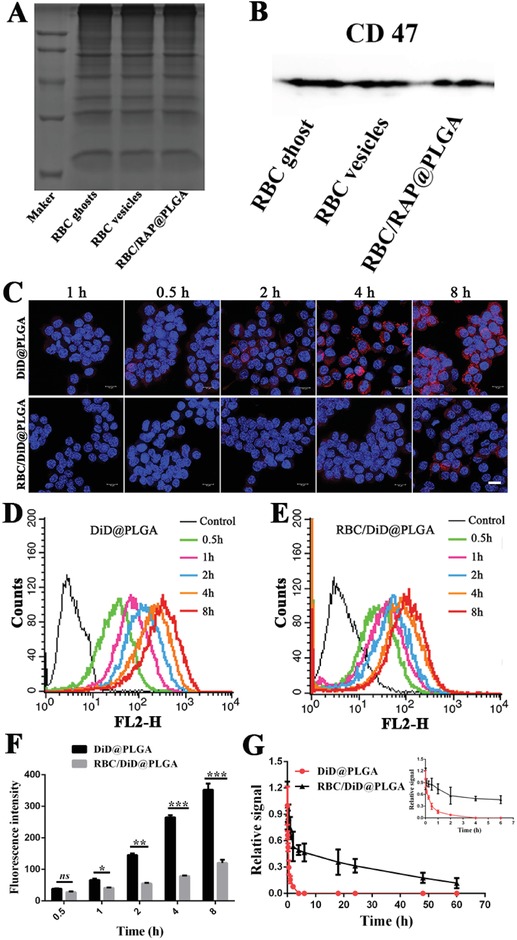
Membrane protein characterization and immune evasive properties in vitro and in vivo. A) Proteins in RBC ghosts, RBC vesicles, and RBC/RAP@PLGA were characterized by polyacrylamide gel electrophoresis. B) Western blot analysis of CD47 in RBC ghost, RBC vesicles, and RBC/RAP@PLGA. C) CLSM images of DiD@PLGA and RBC/DiD@PLGA phagocytosed by RAW264.7 macrophages at different time points (scale bar = 10 µm). Cellular uptake of D) DiD@PLGA and E) RBC/DiD@PLGA in RAW264.7 cells by flow cytometry. F) Quantification of cellular uptake of DiD@PLGA and RBC/DiD@PLGA in RAW264.7 macrophages at different time points (*n* = 3). G) Pharmacokinetic studies of RBC/DiD@PLGA and DiD@PLGA in C57BL/6 mice, (*n* = 5). *p < 0.05, **p < 0.01, and ***p < 0.001. ns, no significance.

Accumulating evidence in the literature suggests that cell membrane proteins are critical for the immune‐evasive function of RBCs.^40^, ^41^ Thus, we first studied if RBC/RAP@PLGA maintained membrane proteins of RBCs. As shown in **Figure** **3**A, SDS‐PAGE suggested that RBC ghosts, RBC vesicles, and RBC/RAP@PLGA were highly consistent in protein bands, revealing that almost all membrane proteins were retained throughout the RBC/RAP@PLGA fabrication. Evidence in the literature suggests that the ability of RBCs to evade macrophage recognition is ascribed to a cooperative contribution of diverse functional membrane proteins on the RBC membrane surface. Among them, CD47, widely expressed on the surface of the RBC membrane, plays a key role in regulating phagocytosis by macrophages by bonding with the SIRP‐α receptor.^41^ Therefore, we tested for CD47 expression on RBC ghosts, RBC vesicles, and RBC/RAP@PLGA using western blot analysis. The results clearly show the presence of CD47 on RBC/RAP@PLGA (Figure 3B).

To study the dynamic uptake of RBC/RAP@PLGA by macrophages, we incubated DiD loaded nanoparticles with RAW264.7 cells and performed time‐lapse studies using confocal laser scanning microscopy (CLSM). As shown in Figure 3C, both DiD@PLGA and RBC/DiD@PLGA were engulfed by macrophages in a time‐dependent manner. However, when compared with DiD@PLGA, phagocytosis of RBC/DiD@PLGA by macrophages was noticeably reduced. We further quantified the distinctive kinetic uptake using flow cytometry (Figure 3D,E) and consistently found that the cellular uptake of DiD@PLGA and RBC/DiD@PLGA were both time‐dependent. However, the internalization signal of RBC/DiD@PLGA was significantly lower than that of DiD@PLGA, as evidenced by 1.6‐, 2.6‐, 3.3‐, and 2.9‐fold decreases at 1, 2, 4, and 8 h incubation, respectively (Figure 3F). The CLSM images and fluorescence activated cell sorting (FACS) analysis collectively suggested that coating nanoparticles with RBC membranes inhibited macrophage‐mediated phagocytosis. The reduced uptake of RBC membrane coated nanoparticles was probably due to the immune‐evasive properties of the RBC membrane proteins.

Compared with unmodified nanoparticles and even the poly(ethylene glycol) modified nanoparticles, RBC membrane‐coated nanoparticles have been reported to show an increased ability to evade phagocytosis by macrophages and systemic clearance, resulting in longer blood‐circulation time.^26^, ^28^, ^29^, ^30^ Subsequently, we utilized C57BL/6 mice as a model to investigate the pharmacokinetics of RBC/DiD@PLGA nanoparticles. After tail vein injection of DiD@PLGA or RBC/DiD@PLGA, blood was drawn at a predetermined time interval from the tail vein to measure the fluorescence intensity. Compared with DiD@PLGA nanoparticles, RBC/DiD@PLGA nanoparticles showed greatly prolonged blood circulation, attributed to the RBC membrane cloaking (Figure 3G). After injecting for 24 and 48 h, the RBC@PLGA and DiD@PLGA nanoparticles exhibited about 31% and 17% overall retention in blood, respectively. However, the bare PLGA nanoparticles (DiD@PLGA) showed negligible signal after 4 h injection, indicating rapid clearance. These results demonstrate that the RBC membrane coated nanoparticles can indeed prolong the blood circulation time.

### 2.3 In Vitro Macrophage Inhibition

Since macrophage cells play an important role in atherosclerosis progression, we examined if RAP loaded nanoparticles inhibit proliferation of RAW264.7 cells, a murine macrophage cell line. Consistent with previous studies, free RAP inhibited macrophage viability in a dose‐dependent manner with an IC50: ≈5 µg mL^−1^ (Figure S7, Supporting Information). Moreover, at the same dose, RAP@PLGA and RBC/RAP@PLGA are comparable in inhibition of macrophage proliferation. The slightly more potent antiproliferative activity of free RAP might be ascribed to the slower RAP release from PLGA.

### 2.4 In Vivo Target Atheroprotective Effect

**Figure 4:**
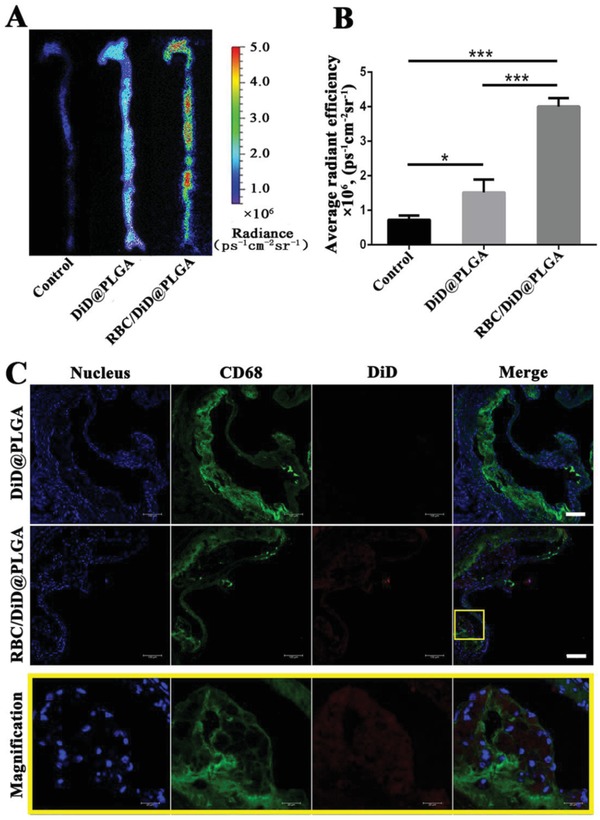
A) The ex vivo fluorescence images of the aorta and B) quantitative data of fluorescence signals accumulated in the aorta of ApoE^−/−^ mice after injection of 5% sucrose (Control), DiD@PLGA, and RBC/DiD@PLGA, *n* = 3, mean ± SD, *p < 0.05 and ***p < 0.001. C) CLSM images of accumulated RBC/DiD@PLGA in atherosclerotic plaques of the aortic root section in ApoE^−/−^ mice. Scale bar = 100 µm.

To study in vivo antiatherosclerosis effects, we first investigated the accumulation of the nanoparticles within atherosclerotic plaques. Atherosclerosis was induced in ApoE^−/−^ mice by feeding with a high‐fat diet (HFD) for 10 weeks. We then administered these mice with DiD@PLGA or RBC/DiD@PLGA through tail vein injection. Aortas were harvested 24 h postinjection. As shown by ex vivo imaging in **Figure** **4**A, RBC/DiD@PLGA clearly accumulated in atherosclerotic plaque areas. Strong fluorescence signals were clearly observed at the aortic arch and abdominal aorta, areas prone to atherosclerosis as a result of abnormal flow patterns. By contrast, the DiD@PLGA group showed significantly lower signals in atherosclerotic plaque compared with the RBC/DiD@PLGA group (Figure 4B). In addition, immunofluorescence histological examination of the aortic root section demonstrated that red fluorescent RBC/DiD@PLGA accumulated in the atherosclerotic plaque, while little DiD@PLGA was observed in the atherosclerotic plaque (Figure 4C). These results demonstrated that the RBC membrane modification enhanced atherosclerotic plaque targeting in vivo. In that case, once RBC/RAP@PLGA nanocomplexes had accumulated in the atherosclerotic lesion, RAP was continuously released from RBC/RAP@PLGA, thereby increasing the local drug concentration to inhibit the proliferation of macrophages and vascular smooth muscle cells (SMCs), and the inflammatory responses in the lesion, and finally significantly attenuating the progression of atherosclerosis. In brief, RBC/RAP@PLGA with targeted drug delivery provided a prerequisite for the subsequent local drug release and efficient atherosclerosis management, resulting from decreased phagocytosis and sustained local drug release, respectively.

**Figure 5:**
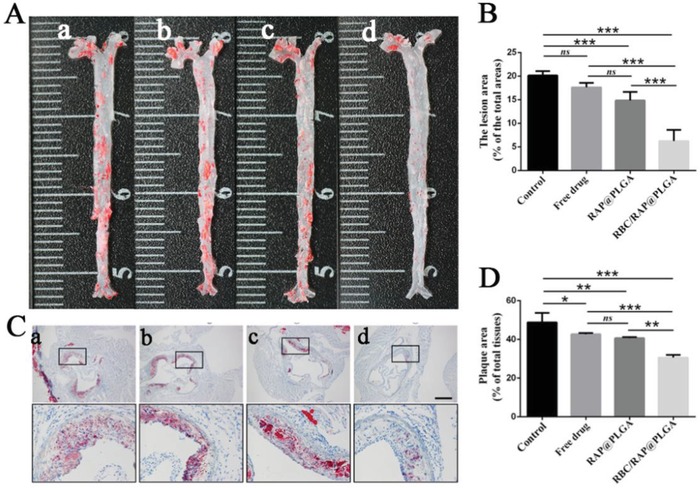
Atherosclerosis treatment by RBC/RAP@PLGA in ApoE^−/−^ mice. A) The en face ORO stained images of aortas from each group (a, Control; b, Free drug; c, RAP@PLGA; d, RBC/RAP@PLGA). B) Quantitative data of the atherosclerotic plaque area. C) ORO‐stained images of aortic roots sections. (a, Control; b, Free drug; c, RAP@PLGA; d, RBC/RAP@PLGA; scale bar = 500 µm). D) Quantitative data of the atherosclerotic plaque area in the aortic root sections. *n* = 5, mean ± SD, *p < 0.05, **p < 0.01, and ***p < 0.001, ns, no significance.

Having confirmed the significantly increased accumulation of RBC membrane‐coated nanoparticles within plaques, we assessed the antiatherosclerosis potential of free RAP, RAP@PLGA, and RBC/RAP@PLGA in ApoE^−/−^ mice. After treatment for one month, we acquired aortas from the aortic arch to the iliac bifurcation and stained with ORO. Control aortas (5% sucrose injection) clearly showed atherosclerotic lesions by en face ORO staining (**Figure** **5**A). Treatment with free drug or RAP@PLGA reduced the lesion area of the plaque. En face quantification of the ORO stained plaque showed that the area ratio of plaque to the whole aorta was decreased from 20.13% to 17.8% and 14.84% after treatment with free drug and RAP@PLGA, respectively (Figure 5B). RBC/RAP@PLGA treatment achieved significantly higher therapeutic efficacy as evidenced by a plaque ratio of 6.24%, confirming the profound inhibition of atherosclerosis progression.

The cross‐sections of ORO‐stained aortic roots further confirmed the extent of plaque in the vascular lumen (Figure 5C). Compared with the value of 47.95% in the control group, the average area ratio of plaque to vascular lumen decreased to 42.42%, 40.48%, and 31.34% after treatment with free drug, RAP@PLGA and RBC/RAP@PLGA, respectively (Figure 5D). These results suggest that RBC/RAP@PLGA can effectively attenuate the progression of atherosclerosis.

**Figure 6:**
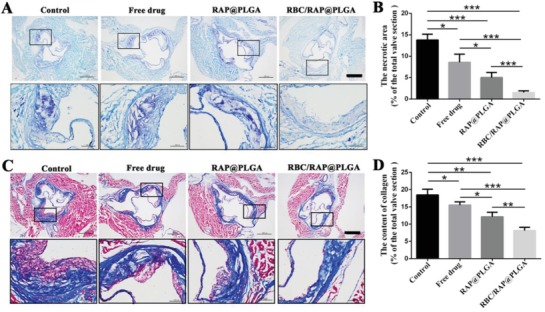
Immunohistochemistry analyses of aortic root sections from ApoE^−/−^ mice after different treatments. A) The images of the necrotic areas stained by Toluidine blue (scale bar = 500 µm). B) Quantitative data of the necrotic areas in the aortic root sections. C) The images of collagen in the plaque areas stained by Masson's trichrome (scale bar = 500 µm). D) Quantitative data of the content of collagen in aortic root sections. *n* = 5, mean ± SD, *p < 0.05, **p < 0.01, and ***p < 0.001, ns, no significance.

Next, we detected the composition of atherosclerotic plaque in aortic root sections by immunohistochemistry staining. The necrotic areas in the aortic roots were detected by Toluidine blue staining. As shown in **Figure** **6**A, the control group exhibited large necrotic areas with substantial cholesterol crystals, indicating advanced lesions. After treating with RBC/RAP@PLGA, the necrotic area was significantly decreased. Quantitative analysis revealed that, compared to the control group, the average necrotic area was decreased to 8.56%, 5.00%, and 1.52% in response to free RAP, RAP@PLGA, and RBC/RAP@PLGA treatment, respectively (Figure 6B). The increased collagen produced by hyperplasia smooth muscle cells leads to the enlargement of the plaque areas, which could further narrow the vascular lumen.^42^ We tested the content of collagen in plaque areas using Masson's trichrome staining (Figure 6C,D). Compared to the control, free RAP, RAP@PLGA, and RBC/RAP@PLGA effectively decreased the content of collagen.

**Figure 7:**
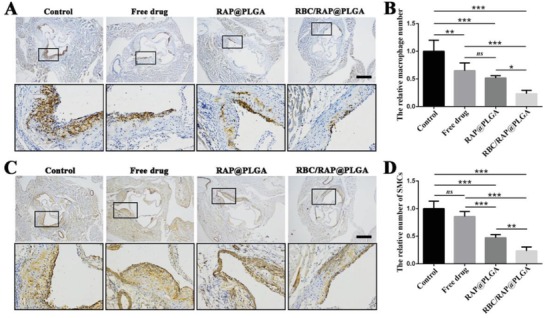
Immunohistochemistry staining of aortic root sections from ApoE^−/−^ mice after different treatments. Representative images of immunohistochemistry staining with antibodies to A) CD68 and C) α‐SMA (scale bar = 500 µm). Quantitative data of B) the relative number of macrophages and D) SMCs in plaque areas of the aortic root sections. *n* = 5, mean ± SD, *p < 0.05, **p < 0.01, and ***p < 0.001, ns, no significance.

Evidence from the literature indicates that macrophages and vascular smooth muscle cells (SMCs) play a key role in the initiation and progression of atherosclerotic inflammation.^42^, ^43^, ^44^ Immunohistochemistry analyses for CD68 (a macrophage marker) (**Figure** **7**A,B) and α‐SMA (a SMC marker) (Figure 7C,D) showed that the number of macrophages and SMCs dramatically decreased in the atherosclerotic plaque area after treating with nanotherapies, particularly after treatment with RBC/RAP@PLGA. These findings confirmed that RAP delivered using nanoparticles could more effectively inhibit the progression of atherosclerosis and suppress luminal narrowing due to the growth inhibition of SMCs and blockade of macrophage infiltration. Matrix metalloproteinases (MMPs), mainly secreted by macrophage‐derived foam cells in atherosclerosis, may well degrade the extracellular matrix within plaque, resulting in plaque rupture. Indeed, the level of MMP‐9 in plaque lesions has been positively associated with the stability of vulnerable plaques.^45^, ^46^ The immunohistochemistry analyses for MMP‐9 showed a notably lower level of MMP‐9 in plaque areas from mice treated with RBC/RAP@PLGA (Figure S8, Supporting Information). This indicated that RBC/RAP@PLGA may stabilize atherosclerotic plaques. Endothelial cells (ECs) play an important role in vascular homeostasis. Dysfunction of the vascular endothelium of the arterial vasculature is an important contributor to the progression of atherosclerotic vascular diseases.^47^ The immunohistochemistry analyses for CD31 (a marker for ECs) showed notable expression of CD31 in the vascular endothelium of the aortas from mice treated with RBC/RAP@PLGA (Figure S9, Supporting Information). This indicated that RBC/RAP@PLGA treatment not only maintains the integrity of the vascular endothelium, but also is unlikely to be toxic to ECs. Collectively, our results demonstrated that RBC/RAP@PLGA more potently attenuated the development of atherosclerotic plaque compared to RAP@PLGA.

### 2.5 Biosafety Assessment

**Figure 8:**
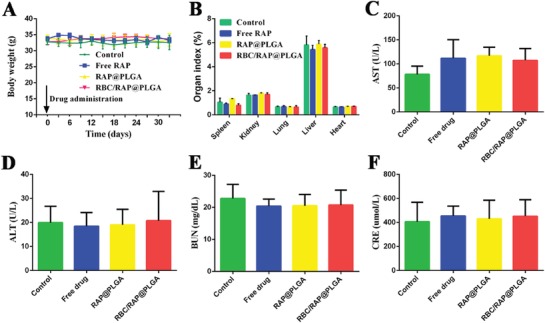
Toxicological evaluations after one month of treatment. A) The body weight of ApoE^−/−^mice during various treatments. B) The organ index. C–F) The biochemical assays of hepatic and kidney functions. AST, aspartate aminotransferase; ALT, alanine aminotransferase; URN, blood urea nitrogen; and CRE, creatinine. *n* = 5, mean ± SD.

**Figure 9:**
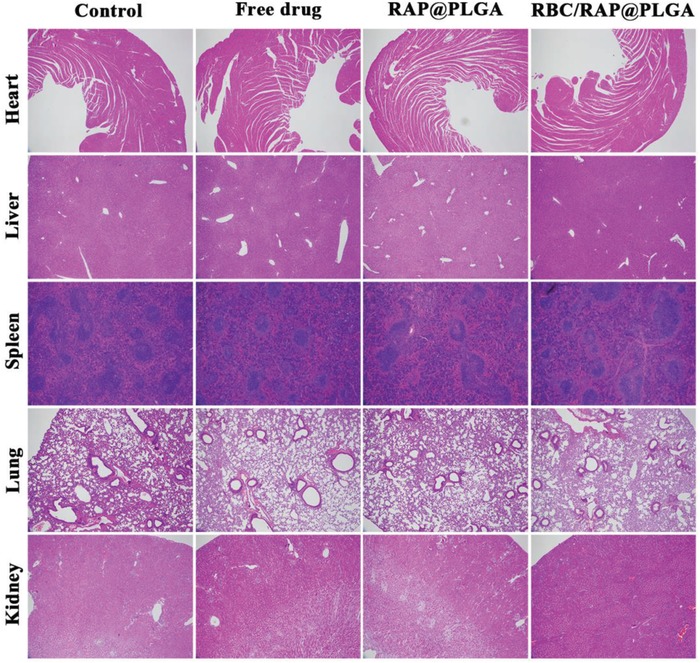
H&E stained images of main organs from mice after various treatments for one month. All the micrographs were acquired at 40× magnification.

To assess biosafety, adverse effects were studied after treatment for one month. There was no significant difference in body weight of mice between the various treated groups (**Figure** **8**A). Moreover, no obvious difference in the organ index of heart, liver, spleen, lung, and kidney was observed, suggesting no significant toxicity to the main organs (Figure 8B). The results of hematoxylin‐eosin (H&E) staining showed that no distinguishable change could be found in the main organs, which further confirmed their biocompatibility (**Figure** **9**). In addition, the blood biochemical assays of alanine aminotransferase (ALT), aspartate aminotransferase (AST), creatinine (CREA), and blood urea nitrogen (UREA) were at normal levels, which indicated that the functions of the liver and kidney were not impaired by the treatment (Figure 8C–F). Routine blood examination implied that levels of RBCs, white blood cells (WBCs), platelets (PLT), and hemoglobin (HGB) did not vary between different mice (Figure S10A–D, Supporting Information). Also, the levels of high density lipoprotein (HDL), low density lipoprotein (LDL), triglyceride (TG), and total cholesterol (TC) did not change significantly during the treatment (Figure S10E–H, Supporting Information). Accordingly, RBC/RAP@PLGA did not induce significant adverse effects in long‐term treatment, indicating its potential as a safe candidate for chronic vascular disease therapy.

## 3 Conclusion

In summary, we developed biomimetic nanocomplexes, consisting of RAP‐loaded PLGA nanoparticles as the core and RBC membranes as a cloak, for effective treatment of atherosclerosis. The resulting biomimetic nanocomplexes (RBC/RAP@PLGA) showed favorable properties including controllable size, negative charge, sustained drug‐release kinetics, effective inhibition of macrophage proliferation in vitro, and long blood circulation in vivo. Mathematical modeling confirmed that drug release from RAP@PLGA and RBC/RAP@PLGA nanoparticles is well‐described by a dissolution–diffusion mechanism. In ApoE^−/−^ mice which induced atherosclerosis by a high‐fat diet, RBC membrane cloaked nanoparticles accumulated in established atherosclerotic plaques. Compared with RAP@PLGA, RBC/RAP@PLGA significantly delayed the progression of atherosclerosis after treatment for one month. Furthermore, the biomimetic nanocomplexes displayed a desirable safety profile without significant side effects, even after long‐term administration in mice. Overall, for the first time, our work has demonstrated the therapeutic advantages of RBC membrane cloaked nanoparticles for the treatment of atherosclerosis. These nanoparticles may be considered as a feasible candidate for a new class of safe and effective targeted drug delivery system for chronic inflammatory disease management.

## 4 Experimental Section

*Materials*: Rapamycin (RAP) and PLGA (MW.90000, 50:50) were purchased from Dalian Meilun Biotechnology Co. Ltd. (Dalian, Chia). 1,19‐dioctadecyl‐3,3,39,39‐tetramethylindodicarbocyanine perchlorate (DiD) was purchased from Biotium Inc. (Fremont, US). DiO, DAPI, and cell total protein extraction kits were supported by Beyotime Institute of Biotechnology (Jiangsu, China). Mouse Glycoprotein ELISA kit was purchased from Wuhan ColorfulGene Biological Technology Co. Ltd. (Wuhan, China).

*Preparation of RBC Vesicles*: RBC vesicles were prepared following a previously reported method with minor modifications.^30^ Whole blood was obtained from C57BL/6 mice (eight weeks) via orbital sinus puncture using EDTA spray‐coated tubes (Labtub, China). Then, the blood was centrifuged at 2500 rpm for 5 min at 4 °C (Centrifuge 5418 R, Eppendorf, Germany) and the serum were carefully removed. The collected RBCs were washed with phosphate buffered saline (PBS, pH = 7.4) three times to remove the residual serum. The washed RBCs were resuspended in dilute 0.25 × PBS (pH = 7.4) containing 0.2 × 10^−3^ m EDTAK2 for 30 min at 4 °C to induce membrane rupture. Subsequently, the RBCs solution was centrifuged at 10 000 rpm for 5 min at 4 °C, and the supernatant was removed. After 3 wash‐centrifugation cycles using PBS, RBC ghosts (RBCG) were collected as the pink precipitation. To obtain RBC vesicles, RBCG were resuspended in water, and then ultrasonicated for 15 min using the sonicator bath (FS30D, 42 kHz, 100 W). The harvested RBC vesicles were stored in water at 4 °C.

*Preparation of Rapamycin‐Loaded PLGA (RAP@PLGA) Nanoparticles*: RAP@ PLGA nanoparticles were prepared by a nanoprecipitation process.^36^ Briefly, RAP (1 mg) and PLGA (10 mg) were dissolved into dimethyl sulfoxide (DMSO) (1 mL). The mixture was precipitated by adding dropwise into 3 mL water with gentle stirring, and further dialyzed using dialysis bag (molecular weight cut‐off, MWCO: 3500 Da) against water to remove the free RAP and DMSO. The solutions of RAP@PLGA nanoparticles were collected, and preserved at 4 °C. To prepare the florescence labeled nanoparticles, 0.1 wt% DiD was loaded in PLGA as per the former method.

*Preparation of RBC Membrane Cloaking RAP@PLGA (RBC/RAP@PLGA) Nanoparticles*: RBC vesicles and RAP@PLGA nanoparticles were fused to prepare RBC/RAP@PLGA by an extrusion method.^38^ Briefly, RBC vesicles (obtained from 500 µL of whole blood) and RAP@PLGA (containing 2 mg of PLGA) were mixed, and ultrasonicated for 2 min using the bath sonicator (FS30D, 42 kHz, 100 W). Then, the mixture solution was extruded using an Avestin mini‐extruder (Avestin, LF‐1, Canada) through a 200 nm polycarbonate porous membrane for 10 times to harvest the RBC/RAP@PLGA.

*Characterization of Nanoparticles*: The size and zeta potential of RAP@PLGA, RBC vesicles and RBC/RAP@PLGA were determined using a Malvern Zetasizer Nano ZS unit (Nano ZS 90, Malvern, UK) with a He‐Ne laser (λ = 633 nm) at a scattering angle of 90° at 25 °C. A drop of RAP@PLGA or RBC/RAP@PLGA nanoparticle solution at a concentration of 150 µg mL^−1^ was dropped onto a copper mesh, and stained by 1% phosphotungstic acid. Subsequently, the morphology of RAP@PLGA and RBC/RAP@PLGA were visually observed using a transmission electron microscope at 200 kV (TEM, JEM‐2100F, JEOL, Japan).

*Characterization of Proteins*: The membrane proteins were characterized by polyacrylamide gel electrophoresis (SDS‐PAGE). The membrane protein of RBC ghosts, RBC vesicle, and RBC/RAP@PLGA were extracted by the cell total protein extraction kits (Beyotime). The extracted membrane proteins were run on 12% SDS‐PAGE gel in a running buffer using BIO‐RAD electrophoresis system at 70 V for 0.5 h and then at 140 V for 1 h. Finally, the SDS‐PAGE gel was stained with SimplyBlue for visualization. CD47 was analyzed by western blot (WB). The RBC ghosts, RBC vesicles, and RBC/RAP@PLGA were lysed with lysis buffer containing 1% phosphatase inhibitors (Beyotime) for 15 min at 4 °C. The lysis solution was centrifuged at 12 000 rpm for 10 min at 4 °C. The supernatant was collected, and the protein concentration was measured using the BCA protein assay kit (Beyotime). The detailed protocol of WB has been described previously.^48^ Briefly, an equal amount of protein from each sample was separated by 12% SDS‐PAGE, and transferred onto a polyvinylidene difluoride membrane (PVDF, Millipore, USA). The transferred PVDF membranes were blocked with 5% milk, and then incubated with primary antibodies against CD47 (Anti‐CD47 antibody, ab175388, abcam) for 12 h at 4 °C. Finally, the membranes were incubated with HRP‐conjugated secondary antibodies and observed by ChemiDoc‐ XRS imaging system (Bio‐Rad).

*Identification of Membrane Orientation of RBC/RAP@PLGA*: To identify the membrane orientation of RBC/RAP@PLGA, the glycoprotein content in the RBC/RAP@PLGA nanoparticles was quantified as previously reported.^38^, ^39^ Briefly, 1 mL of solution of RBC/RAP@PLGA (1 mg mL^−1^) was incubated with 5 µg of trypsin at room temperature for 2 h to initiate trypsinization. Then, the samples were centrifuged at 8000 rpm for 5 min, and the supernatant was collected to quantify glycoprotein using a Mouse Glycoprotein ELISA Kit following the manufacturer's instructions.

*Drug Loading and In Vitro Drug Release Study*: To calculate the loading efficiency of RAP, RAP@PLGA lyophilized powder was dissolved in DMSO, and the absorbance measured by UV–vis spectrophotometer (DU730, Beckman Coulter) at 280 nm. According to the pre‐established standard curve of RAP in DMSO, the drug loading efficiency (LE) and drug encapsulation efficiency (EE) were calculated as the following equations

(1) $$\mathrm{LE}\;\left(\%\right)=\frac{M_{\mathrm{RAP}}}{M_{\mathrm{PLGA}}+M_{\mathrm{RAP}}}\;\times \;100\%$$

(2) $$\mathrm{EE}\;\left(\%\right)=\frac{M_{\mathrm{RAP}}}{M_{\mathrm{added}}}\;\;\times \;100\%\;$$

in which *M*_RAP_ is the mass of RAP loaded in the nanoparticles, *M*_PLGA_ is the mass of polymer in the formulation and *M*_added_ is the mass of added RAP.

To study the drug release profile in vitro, RAP@PLGA and RBC/RAP@PLGA nanoparticle solutions (1 mg mL, 1 mL) were added to disposable dialysis cups (Slide‐A‐Lyzer MINI Dialysis Units, MWCO: 3500 Da, Thermo Scientifc) in PBS (10 mL). At different time points, the external drug release buffers were collected and an equivalent amount of PBS was added. The cumulative amount of RAP released was quantified by high performance liquid chromatography (HPLC).

*Mathematical Modeling of In Vitro Drug Release*: A mathematical model was developed to describe RAP release from the RAP@PLGA and RBC/RAP@PLGA nanoparticles. Given that RAP was extremely poorly soluble, it was proposed that dissolution in the release medium would be an important factor in determining the release rate. A homogeneous distribution of drug encapsulated in the PLGA initially at some concentration *B* was assumed and it was noted that there was no significant degradation of PLGA over the 72 h studied. Further, a radially symmetric model was assumed for the nanoparticles of radius *R* and the spatial and temporal coordinates were denoted by *r* and *t*, respectively. When exposed to the PBS release medium, the nanoparticles become wetted and a dissolution process ensues, converting immobile drug (of concentration *b*(*r*, *t*)) to dissolved drug (of concentration *c*(*r*, *t*)). When dissolved, the drug was able to diffuse through the nanoparticle with diffusion coefficient *D*. Following previous work,^49^ the dissolution process was modeled through a nonlinear reaction whereby dissolution occured at some rate *Kb*(*r*, *t*)^2/3^ (*K* constant) and in proportion to the distance between the dissolved drug concentration and the maximum dissolved drug concentration, denoted by *S*, the solubility. The exponent 2/3 arose based on the assumption that the surface area of undissolved drug was proportional to the volume (for more details, the reader is referred to Frenning^50^). The model was then given by

(3) $$\frac{\partial b}{\partial t}=\;-Kb^{2/3}\left(S-c\right),\;0<r<R,\;t>0$$

(4) $$\frac{\partial c}{\partial t}=\;D\left(\frac{\partial^{2}c}{\partial r^{2}}+\frac{2}{r}\frac{\partial c}{\partial r}\right)\;+Kb^{2/3}\left(S-c\right),\;0<r<R,\;t>0$$

(5) $$\frac{\partial c}{\partial r}=\;0,\;r\;=\;0,\;t>0$$

(6) c = 0, r = R, t>0

(7) b = B, c = 0, 0<r<R, t = 0

The boundary conditions given by Equations (5) and (6) represented symmetry and sink conditions, respectively. The model given by Equations (3), (4), (5), (6), (7) was utilized to describe drug release from the RAP@PLGA nanoparticles. In order to model drug release from the RBC/RAP@PLGA nanoparticles, it was supposed that the RBCs act as a thin membrane and, therefore, the boundary condition (6) was modified to obtain

(8) $$-D\;\frac{\partial c}{\partial r}=\;P\;c,\;r\;=\;R,\;t>0$$

where *P* provides a measure of the membrane resistance.

The model represents a set of nonlinear coupled partial differential equations. To solve the equations, an approach similar to the method we previously described was adopted.^49^ Briefly, the equations were discretized in space and then Matlab's ODE45s solver for stiff problems was used to solve the resulting set of coupled ordinary differential equations. For numerical convenience, the model was non‐dimensionalized by letting

(9) $$\bar{t}=\frac{t}{T_{D}}\;,\;\bar{r}=\frac{r}{R}\;,\;\bar{c}=\frac{c}{B},\;\bar{b}=\frac{b}{B},\;\;\bar{S}=\frac{S}{B}$$

where *T*_D_ = *R* ^2^/*D* was the timescale for diffusion. The resulting nondimensional model for RAP@PLGA (see Supporting Information) consisted of only two nondimensional parameters: i) the normalized solubility $\bar{S}$, and ii) the Damköhler number *Da* = *KB* ^2/3^ *R* ^2^/*D*, which characterized the rate of dissolution to the rate of diffusion. The nondimensional model for RBC/RAP@PLGA contained a further nondimensional parameter, Γ = *R* *P*/*D*, which characterized the membrane resistance.

*Co‐Localization Study*: RAW 264.7 cells were cultured in DMEM medium containing 10% fetal bovine serum (FBS) at 37 °C with 5% CO_2_. Thereafter, 100 µg of RBC/DiD@PLGA was added to RAW 264.7 cells, in which the RBC membrane of RBC/DiD@PLGA was labeled by DiO. After incubation for an additional 2 h, the cells were washed with PBS three times, and fixed with paraformaldehyde (4% in PBS) for 30 min at room temperature. Then, the nucleus of RAW 264.7 cells were stained with 4′,6‐diamidino‐2‐phenylindole (DAPI). The confocal laser scanning microscopy (SP8, Leica, Germany) images of the cells were obtained under 405, 488, and 644 nm filters, respectively.

*Nanoparticles Uptake by Macrophages*: RAW264.7 cells were seeded in 12‐well plates at a density of 1 × 10^5^ cells per well in 1 mL of DMEM medium containing 10% FBS, and cultured at 37 °C with 5% CO_2_ for 24 h. 100 µg of DiD@PLGA and RBC/DiD@PLGA were added. After incubation for different times, the cells were washed with PBS, and fixed with paraformaldehyde (4% in PBS). The nuclei of the cells were stained with DAPI. The cells were observed using CLSM. For cell uptake quantification, RAW264.7 cells without DAPI staining were digested, and the fluorescent intensity of these samples was subsequently measured by fluorescence activated cell sorting.

*Inhibition of Proliferation of Macrophages In Vitro*: RAW264.7 cells were seeded in a 96‐well plate (10^5^ cells per well), and cultured in DMEM medium containing 0.5% FBS for 12 h. Then, the cells were incubated with various doses of free RAP, RAP@PLGA, and RBC/RAP@PLGA for 24 h, respectively. The cell viability was quantified by CCK‐8 assay.

*Animals*: Male C57BL/6 mice and male apolipoprotein E‐deficient (ApoE^−/−^) mice (25–30 g, eight‐week old) were obtained from the Third Military Medical University in Chongqing, China. All the animal care and experimental protocols were carried out with review and approval from the Laboratory Animal Welfare and Ethics Committee of the Third Military Medical University.

*In Vivo Pharmacokinetics Study*: The experiments utilized adult male C57BL/6 mice weighing 25 ± 2 g as the in vivo model. To study the half‐life of RBC/DiD@PLGA in circulation, 150 µL of DiD@PLGA or RBC/DiD@PLGA was injected into the mice through the tail vein. 20 µL of blood was collected at 1, 5, 15, 30 min, and 1, 2, 4, 6, 18, 24, 48, and 60 h after injection. The blood samples were diluted with 40 µL PBS contained 0.2 × 10^−3^ m EDTA2K in 96‐well plates, and the fluorescence intensity was measured by fluorescence microplate reader (SpectraMax Gemini EM, USA).

*Accumulation of Nanoparticles in Atherosclerotic Plaques of ApoE^−/−^ Mice*: ApoE^−/−^ mice were fed with a high fat diet (HFD) for 10 weeks. 150 µL DiD@PLGA or RBC/DiD@PLGA was injected through the tail vein. After 24 h, mice were euthanized, perfused with PBS containing 4% paraformaldehyde and heparin sodium, and the aorta was isolated for imaging and fluorescence quantification using a Xenogen IVIS 200 system. The aortas of 5% sucrose‐treated mice were used as the control group to subtract the tissue auto‐fluorescence. Immunofluorescence staining of the cross‐sections of the aortic roots was performed as previously described. The frozen sections of carotid roots were incubated with 5% serum. Then, the sections were incubated with anti‐CD68 antibody overnight at 4 °C, followed by Donkey anti‐rabbit IgG H&L for 2 h at room temperature. Samples were stained with DAPI to show the cell nucleus. The sections were observed by CLSM.

*Treatment of Atherosclerosis in ApoE^−/−^ Mice*: Twenty ApoE^−/−^ mice were randomized into 4 groups (5 mice per group), and given the HFD for 10 weeks. Then, the mice were subjected to the different treatments for one month. The mice injected with 5% sucrose served as the control group, while the other three groups were treated with either free drug, RAP@PLGA or RBC/RAP@PLGA at a dose of 0.7 mg kg^−1^ of RAP every three days via tail vein injection. The body weight of mice was monitored during the treatment.

*Quantitative Analysis of Atherosclerotic Plaques after Treatment*: After treatment for 30 d the aortas, from the heart to the iliac bifurcation, from ApoE^−/−^ mice were harvested. Aortas were fixed by perfusion with paraformaldehyde (4% in PBS). After removing the periadventitial tissue, aortas were dissected longitudinally, and then stained with Oil Red O (ORO) to quantify the plaque area. The extent of atherosclerotic plaque at the aortic root was also determined by ORO staining. Quantitative analysis of atherosclerotic plaque areas was performed using Nis‐Elements BR 3.2 software (Nikon, Japan).

*Histology and Immunohistochemistry Staining of the Aortic Root*: The aortic roots were fixed with paraformaldehyde (4% in PBS) for 1 h, and then prepared to paraffin sections. After deparaffinizing, Masson's trichrome and Toluidine blue staining were used to quantify the content of collagen and the necrotic core, respectively. For immunohistochemistry analysis, the activity of the endogenous peroxidase was inhibited by immersion into 3% hydrogen peroxide and 100% methanol for 20 min. Then, the sections were blocked with 5% bovine serum albumin in PBS for 60 min. Antibodies to CD68, α‐smooth muscle actin (α‐SMA), matrix metalloproteinase‐9 (MMP‐9) or CD31 were incubated for quantification of macrophage, MMP‐9, smooth muscle cells (SMCs) and endothelial cells (ECs) respectively. Quantitative analysis of histology and immunohistochemistry were performed using the Nis‐Elements BR 3.2 software (Nikon, Japan). Sections of the main organs including heart, liver, spleen, lung, and kidney were also analyzed by hematoxylin‐eosin (HE) staining.

*Complete Blood Biochemistry and Routine Analysis*: Blood was collected in EDTA2K spray‐coated tubes after treatment for one month, and immediately analyzed using an automated hematology analyzer (Sysmex KX‐21, Sysmex Co., Japan). The concentrations of alanine aminotransferase, aspartate aminotransferase, creatinine, blood urea nitrogen , high density lipoprotein, low density lipoprotein, triglyceride, and total cholesterol in plasma from different treatments were quantified by an automated analyzer platform (Roche Cobas C501, Roche Co., Switzerland).

*Statistical Analyses*: The data obtained are reported as the mean ± standard deviation in this study. GraphPad Prism Version 6.0 software (GraphPad, USA) was used for the statistical analysis. One‐way analysis of variance (ANOVA) by Tukey's test was used to reveal differences among the groups. The difference significance levels were set at *p < 0.05, **p < 0.01, and ***p < 0.001.

## Conflict of Interest

The authors declare no conflict of interest.

## Supporting information

Supplementary
