# Supplementary material for: Biomimetic Nanotherapies: Red Blood Cell Based Core–Shell Structured Nanocomplexes for Atherosclerosis Management
Source: Adv Sci (Weinh). 2019 Apr 24;6(12):1900172. doi: 10.1002/advs.201900172 (PMC6662054; doi:10.1002/advs.201900172)
Supplement: Supplementary file 1 — Supplementary [file ADVS-6-1900172-s001.pdf]

Copyright WILEY-VCH Verlag GmbH & Co. KGaA, 69469 Weinheim, Germany, 2019.

ADVANCED SCIENCE
Open Access

Supporting Information

for *Adv. Sci.,* DOI: 10.1002/advs.201900172

Biomimetic Nanotherapies: Red Blood Cell Based Core–Shell Structured Nanocomplexes for Atherosclerosis Management

Yi Wang, Kang Zhang, Xian Qin, Tianhan Li, Juhui Qiu, Tieying Yin, Junli Huang, Sean McGinty, Giuseppe Pontrelli, Jun Ren, Qiwei Wang, Wei Wu, and Guixue Wang**

Supporting Information

Biomimetic Nanotherapies: Red Blood Cell Based Core-Shell Structured Nanocomplexes for Atherosclerosis Management

Yi Wang[a], Kang Zhang[a], Xian Qin[a], Tianhan Li[a], Juhui Qiu[a], Tieying Yin[a], Junli Huang[a], Sean McGinty[b], Giuseppe Pontrelli[c], Jun Ren[d], Qiwei Wang[e], Wei Wu[a,*], Guixue Wang[a,*]

[a]Key Laboratory for Biorheological Science and Technology of Ministry of Education, State and Local Joint Engineering Laboratory for Vascular Implants, Bioengineering College of Chongqing University, Chongqing, 400030, China.

[b]Division of Biomedical Engineering, University of Glasgow, UK.

[c]Istituto per le Applicazioni del Calcolo - CNR, Via dei Taurini 19, 00185, Roma, Italy

[d]Department of Radiation Oncology, Massachusetts General Hospital, Harvard Medical School, Boston MA 02114, USA

[e]Department of Cancer Biology, Dana-Farber Cancer Institute and Department of Biological Chemistry and Molecular Pharmacology, Harvard Medical School, Boston MA 02115, USA

Email: wanggx@cqu.edu.cn (Guixue Wang), david2015@cqu.edu.cn (Wei Wu).

Table S1

Loading and encapsulation efficiency of Rapamycin (RAP) into PLGA nanoparticles.

	μg/mg	Drug Loading Efficiency (%)	Drug Encapsulating Efficiency (%)
RAP@PLGA	77.9±1.4	7.79	84.5

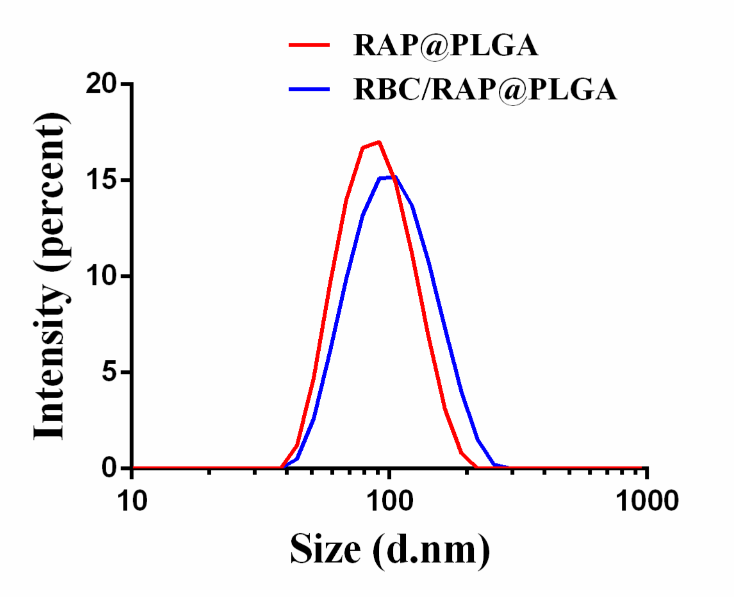

Figure S1. The size distributions of RAP@PLGA and RBC/RAP@PLGA characterized by DLS.

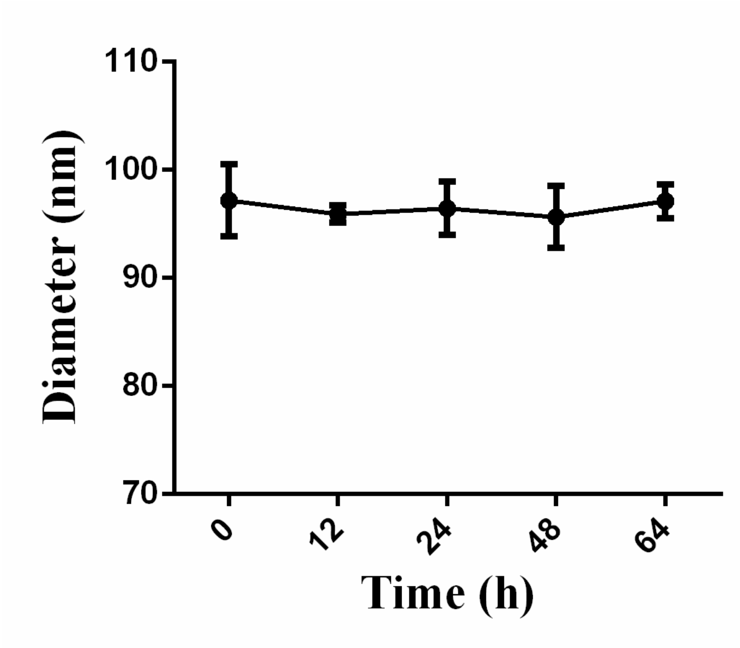

Figure S2. Size change tendency of RBC/RAP@PLGA (*n* = 3).

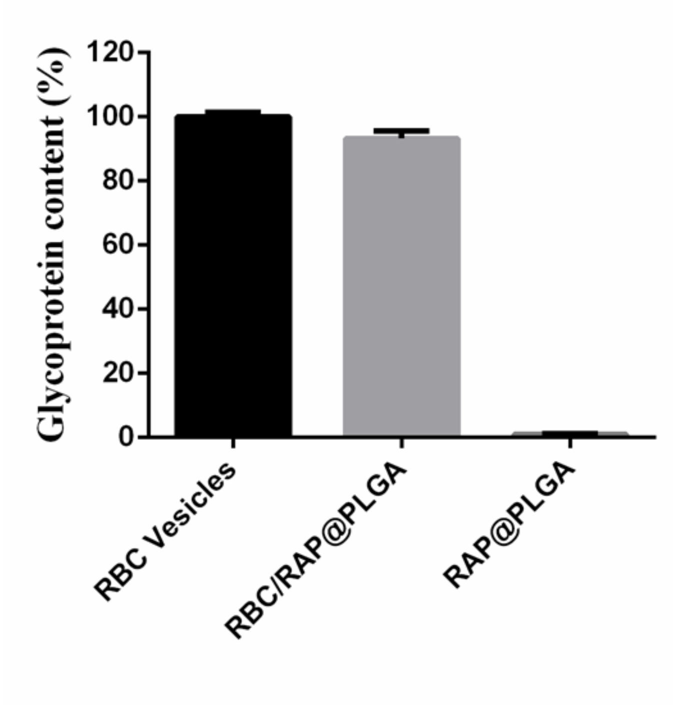

Figure S3. Confirmation of the orientation of RBC membranes on the nanoparticles by comparing the relative glycoprotein content of the RBC vesicles, RBC/RAP@PLGA and RAP@PLGA (n = 3).

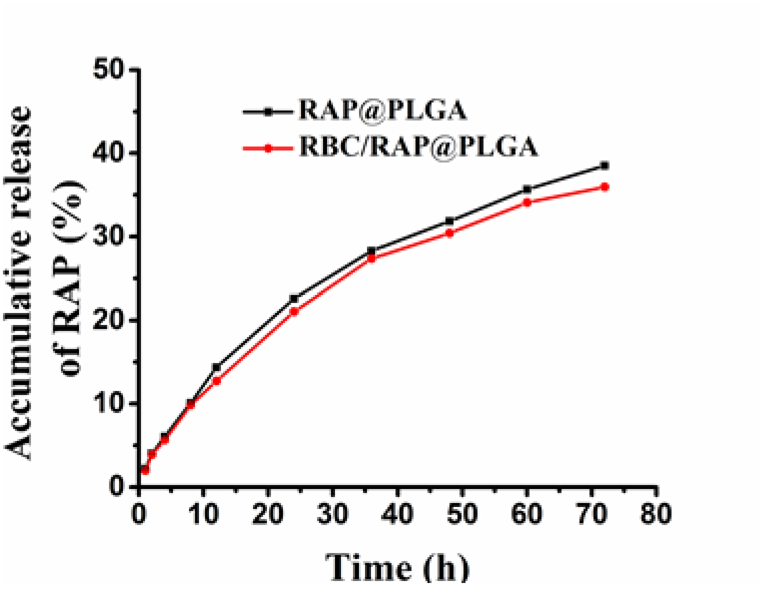

Figure S4. *In vitro* release profiles of RAP@PLGA and RBC/RAP@PLGA in PBS (pH 7.4) (n = 3).

Mathematical Modeling of *in Vitro* Drug Release – Non-Dimensional Model

Letting

$$\bar{t} = \frac{t}{T_D}, \qquad \bar{r} = \frac{r}{R}, \qquad \bar{c} = \frac{c}{B}, \qquad \bar{b} = \frac{b}{B}, \qquad \bar{S} = \frac{S}{B}, \qquad (S1)$$

we non-dimensionalize the model given by equations (3-7) of the main text to obtain

$$\frac{\partial \bar{b}}{\partial \bar{t}} = -Da\, \bar{b}^{2/3}(\bar{S} - \bar{c}), \quad 0 < \bar{r} < 1, \qquad \bar{t} > 0, \qquad (S2)$$

$$\frac{\partial \bar{c}}{\partial \bar{t}} = \left(\frac{\partial^2 \bar{c}}{\partial \bar{r}^2} + \frac{2}{\bar{r}}\frac{\partial \bar{c}}{\partial \bar{r}}\right) + Da\, \bar{b}^{2/3}(\bar{S} - \bar{c}), \quad 0 < \bar{r} < 1, \qquad \bar{t} > 0, \qquad (S3)$$

$$-\frac{\partial \bar{c}}{\partial \bar{r}} = 0, \quad \bar{r} = 0, \;\; \bar{t} > 0, \quad \text{(S4)}$$

$$\bar{c} = 0, \quad \bar{r} = 1, \;\; \bar{t} > 0, \quad \text{(S5)}$$

$$\bar{b} = 1, \quad \bar{c} = 0, \;\; 0 < \bar{r} < 1, \;\; \bar{t} = 0, \quad \text{(S6)}$$

where $Da = KB^{2/3}\, R^2/D$. For the case of RBC/RAP@PLGA, the boundary condition (S5) is replaced with

$$\frac{\partial \bar{c}}{\partial r} = -\Gamma\, \bar{c}, \;\; \bar{r} = 1, \qquad \bar{t} > 0, \qquad (S7)$$

where $\Gamma = R\, P/D$ characterises the membrane resistance.

For the purposes of the numerical computations, the spatial domain was divided into 100 equally spaced strips and the time step was optimized by the ODE45s program.

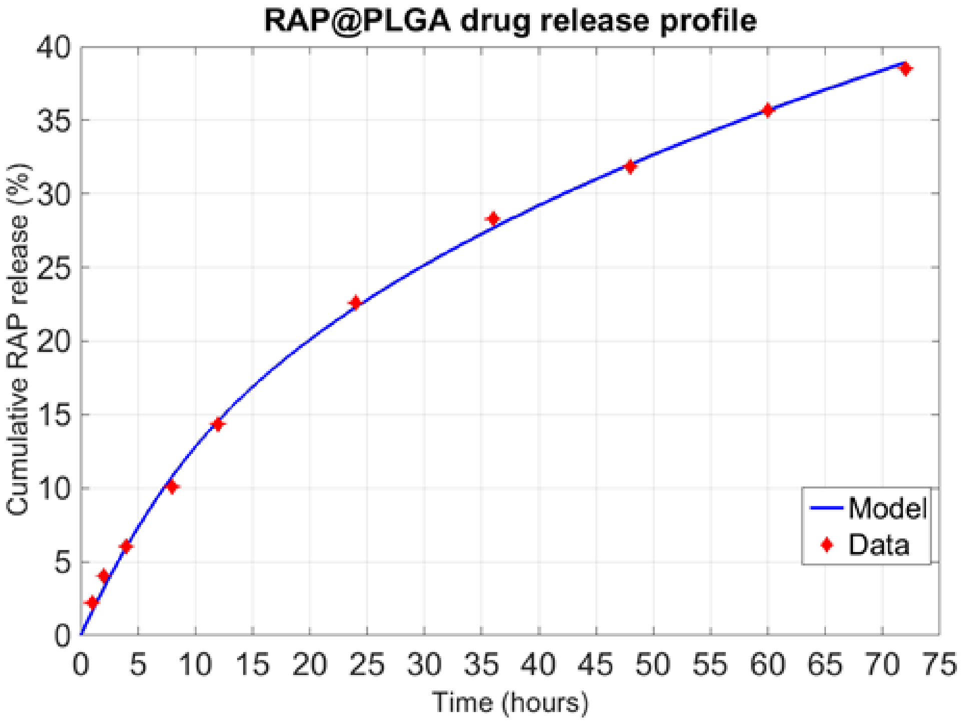

Figure S5. Comparison between the dissolution-diffusion mathematical model and the experimental data for drug release from RAP@PLGA nanoparticles. Using the least squares method, the best fit was found when $Da = 1396$ and $\bar{S} = 1.66 \times 10^{-5}$.

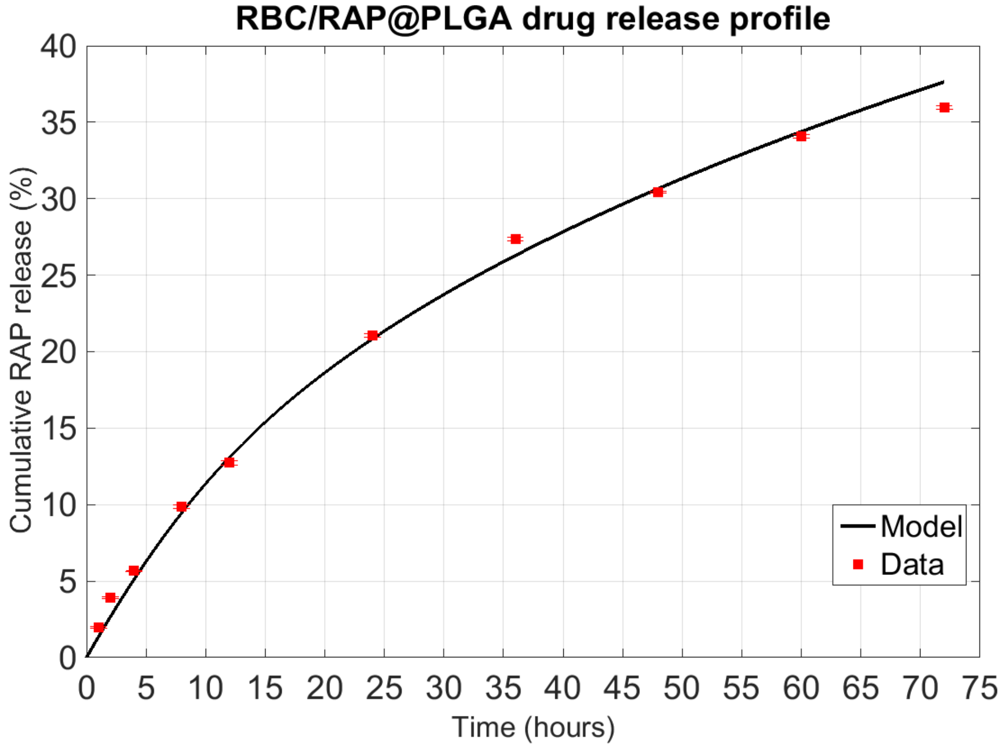

Figure S6. Comparison between the dissolution-diffusion mathematical model and the experimental data for drug release from RBC/RAP@PLGA nanoparticles. Using the same Damköhler number of $Da = 1396$ and normalized solubility of $\bar{S} = 1.66 \times 10^{-5}$ as the RAP@PLGA case, the best fit was found when the membrane resistance $\Gamma = 173$.

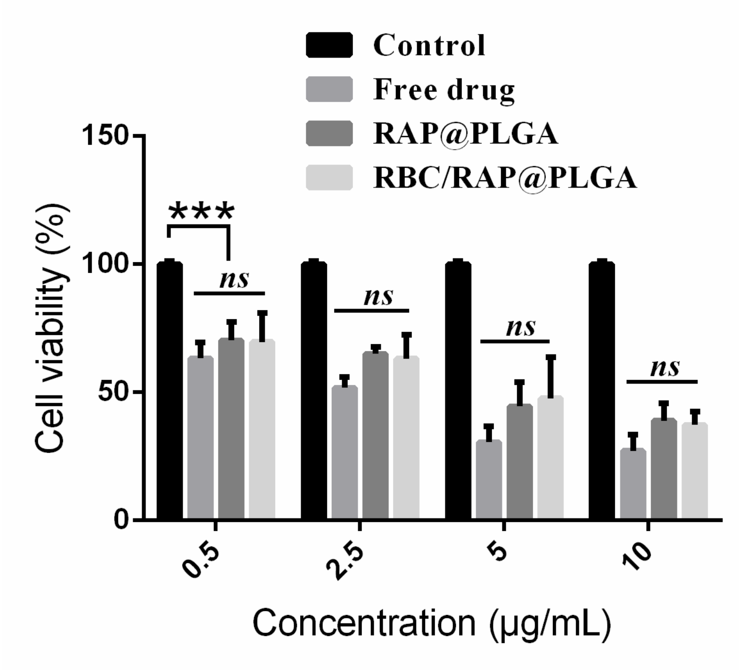

Figure S7. Proliferation of RAW264.7 cells after 24 h of incubation with free RAP, RAP@PLGA and RBC/RAP@PLGA at different concentration (n = 5).

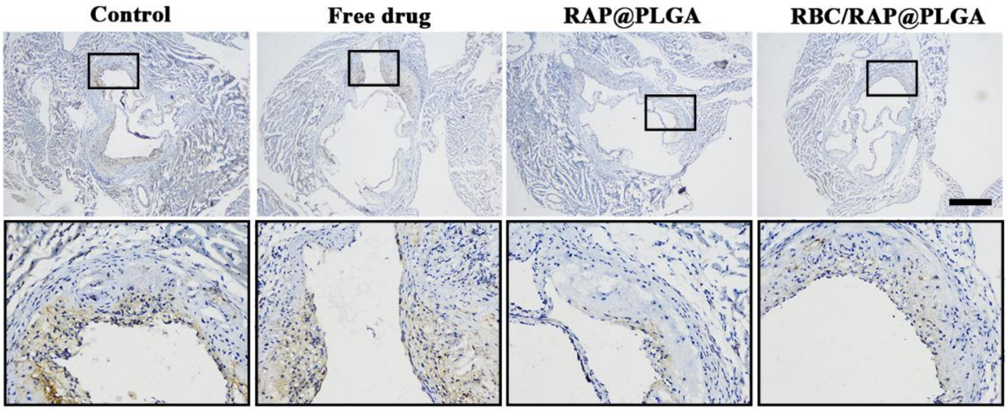

Figure S8. Immunohistochemistry analysis on the sections of aortic roots from ApoE$^{-/-}$ mice post different treatments. Representative photographs of immunohistochemistry staining with antibodies to MMP-9 (scale bar = 500 μm).

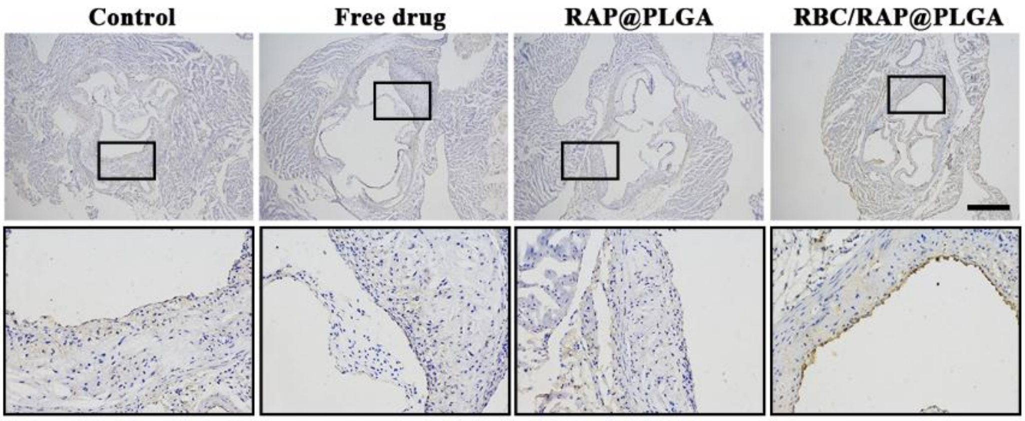

Figure S9. Immunohistochemistry analysis on the sections of aortic roots from ApoE$^{-/-}$ mice post different treatments. Representative photographs of immunohistochemistry staining with antibodies to CD31 (scale bar = 500 μm).

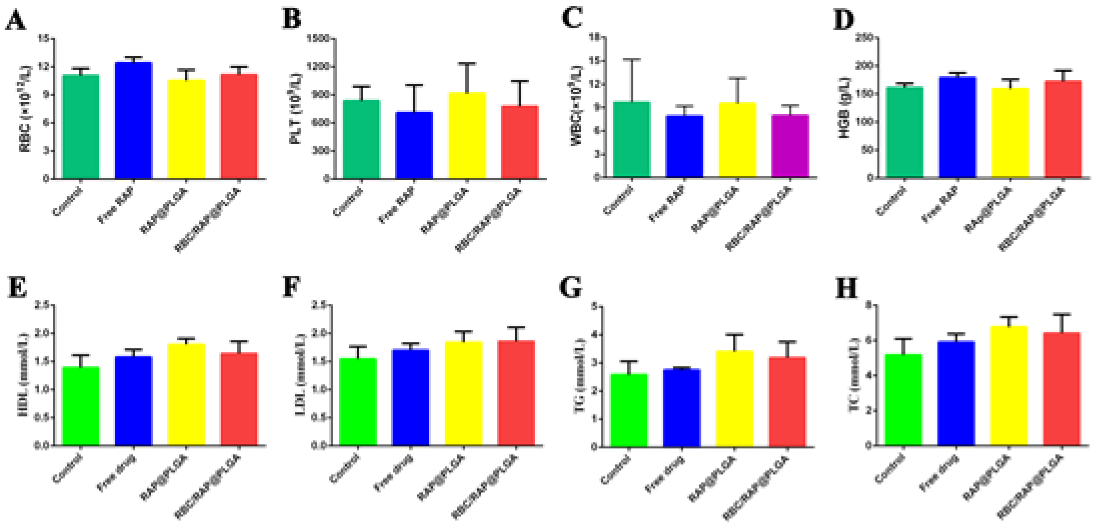

Figure S10. Typical hematological parameters (A-B). RBC, red blood cell; WBC, white blood cell; PLT, platelet; and HGB, hemoglobin. Four items of blood lipid tests (F-H). TC, the serum total cholesterol; TG, triglyceride; LDL, low density lipoprotein cholesterol; HDL, high density lipoprotein cholesterol (*n*=5).
